# Urbanization and depressive symptoms among middle-aged and older adults in China

**DOI:** 10.3389/fpubh.2022.1086248

**Published:** 2022-12-23

**Authors:** Chenlu Hong, Xiaoxiao Xiong, Jun Li, Xin Ning, Dawei Qi, Yingkai Yang, Yating Liu, Yanan Luo

**Affiliations:** ^1^Department of Global Health, School of Public Health, Peking University, Beijing, China; ^2^National Academy of Innovation Strategy, Beijing, China; ^3^School of Nursing, Peking University, Beijing, China; ^4^School of Nursing, Kunming Medical University, Kunming, Yunnan, China; ^5^Central Health Center of Qingyundian Town, Beijing, China; ^6^Institute for Global Health and Development, Peking University, Beijing, China

**Keywords:** urbanization, depressive symptoms, mechanism, middle-aged and older adults, the China Health and Retirement Longitudinal Study

## Abstract

**Aims:**

Urbanization plays an important role in individuals' health. However, it is difficult to isolate healthy migrant effect between urbanization and health. This study examined the effects of urbanization on depressive symptoms and its possible pathways among Chinese middle-aged and older adults independent of the influence of health-selective migration.

**Methods:**

Using the baseline survey of the China Health and Retirement Longitudinal Study, this study compared the depressive symptoms among three groups (urbanized rural residents, rural non-migrants and urban non-migrants). The 10-item Center for Epidemiologic Studies Depression Scale (CESD-10) short form was used to measure depressive symptoms. Logistic regression models and Structural Equation Model (SEM) were applied to examine the association between urbanization and depressive symptoms and the corresponding potential mechanisms.

**Results:**

Our final sample contained 11,156 respondents with an average age of 58.91 (SD = 9.48), with 5,142 males (46.09%) and 6,014 females (53.91%). Compared with urbanized rural residents, rural residents were more likely to have depressive symptoms (OR = 1.19, 95% CI = 1.07, 1.32), and urban residents were associated with a decreased risk of depressive symptoms (OR = 0.81, 95% CI: 0.70, 0.94). A large proportion of the association between urbanization and depressive symptoms were mainly mediated by social participation, income and living conditions.

**Conclusions:**

Planned urbanization had an independent impact on decreased depressive symptoms. Improvements in social participation, income and living conditions are the main drivers behind this relationship. Additionally, urbanization compensates for the negative impact of depressive symptoms from disadvantaged early life conditions, but it cannot eliminate the gap between urbanized rural people and urban non-migrants.

## 1. Introduction

Depression is a major public health concern. According to WHO data, approximately 280 million people suffer from depression. The disease burden of depression ranks 13th among the leading causes of disability-adjusted life years (DALY) (1). It will increase the risk of adverse health outcomes, including cardiovascular diseases, dementia, cognitive impairment and falls (2–4) and cause a huge economic burden (5), with an annual medical cost of $326 billion in the US in 2020 (6) and €118 billion in Europe in 2005 (7). Depressive symptoms are very common among middle-aged and older adults, presenting in up to one-third of older adults (8), and serve as an early indicator of depression (9). In China, the lifetime prevalence of depressive symptoms in adults over 50 years old is ~4.1% (10), which has hurt health systems (1), with an annual medical expense of RMB 4.4 billion in urban areas (11). In the context of aging in China, where the population aged 65 and over is projected to account for 26.1% by 2050, it is important to study the prevention and intervention of depressive symptoms to improve the quality of life of middle-aged and older adults.

The health effect of urbanization is controversial (12). On the one hand, it provides protective health opportunities, especially for people with depression, such as better living conditions, infrastructure development and access to health care (13–15). On the other hand, it is also associated with a range of factors that can further increase depression risk due to rapid and unplanned urbanization progress, including poor quality of healthcare, economic pressure and environmental hazards (16, 17). In the past decade, China has been rapidly urbanized, with a proportion of 49.68% in 2010 to 63.89% in 2020, far exceeding that of other countries. Different from other low-income and middle-income countries, China's rapid urbanization is mainly encouraged by governments (16, 18). Therefore, this process avoids the negative impact of urbanization, such as slums in developing countries in the progress of urbanization (19), which is partly due to the expansion of housing and infrastructure brought about by urban planning (20). Additionally, due to the improvement of socioeconomic conditions, infrastructures, healthcare services, and social inclusion in urban areas (13, 14), this process may provide benefits to health promotion.

China's urbanization is usually accompanied by large rural-to-urban migration, including voluntary migration and forced urbanization (20). The drivers of voluntary migration can be described as economic, social and environmental push or pull factors such as better job opportunities or housing conditions in urban areas (21). As a result, those migrants are selected individuals who tend to be healthier according to the hypothesis of health-selective migration. Furthermore, according to salmon bias, migrants with poor health status are more likely to return to their destinations (22). For these reasons, it remains unclear whether the health benefits observed in urban areas are due to urbanization or simply the result of health-selective migration. Urbanization in China can also include forced urbanization in urbanized villages, where the whole village territory is converted to urban land, and the so-called forced upstairs farmers are involuntarily relocated from traditional scattered houses to urban multistory housing buildings by the government (23, 24). Therefore, urbanization can be studied as an exogenous variable since these population groups do not experience migration in urbanized villages. In light of the above, planned urbanization in China provides us with a special opportunity to study the causal relationship between urbanization and depressive symptoms. To isolate the effect of health-selective migration or salmon bias, this study identifies a population in China. This population is transformed from local villagers to urban citizens without being influenced by migration, sharing early life experiences with the rural population that has stayed in rural areas all their lives and their later years with urban residents that have resided in cities throughout their lives.

Although previous studies have documented significant associations between urbanization and depression (13, 25), most of these related studies are mainly regional surveys, and few of them detect the underlying mechanism through which urbanization influences depressive symptoms. Additionally, it is difficult to rule out the effect of health-selective migration or salmon bias in much of the research on urbanization and improved health (16, 22). To bridge these gaps, this study aims to investigate the association between urbanization and depressive symptoms and further detect the potential causal pathways between them by using large nationally representative data from the China Health and Retirement Longitudinal Study (CHARLS) among middle-aged and older adults in China. This study may help to understand the impact of urbanization independent of health-selective migration on depressive symptoms and provide references for the early prevention and intervention of mental health promotion for low-income and middle-income countries in their urbanization progress. Based on prior screening and background, we hypothesize two empirical predictions under the hypothesis that urbanization is good for depressive symptoms as follows:

Hypothesis 1. The health of the in urbanized rural residents will be (a) better than that of the rural residents, and (b) worse than that of the urban residents.

Hypothesis 2. (c) More engagement in social participation, (d) more utilization of healthcare, (e) higher individual income, and (f) better living conditions in urban areas will contribute to this advantage.

## 2. Materials and methods

This study used data from the China Health and Retirement Longitudinal Study (CHARLS), which is a nationally representative survey in China. The objectives of this survey were to provide information about demographic characteristics, health status and functioning, health care and insurance, and socioeconomic conditions. Face-to-face computer-assisted personal interview (CAPI) was conducted every 2 years. Samples were obtained by the probability-proportional-to-size (PPS) sampling technique to ensure the representativeness of the sample. In the first stage, all counties (except Tibet) were stratified by region, urbanity and GDP per capita. Primary sampling units (PSUs) were chosen among each selected county using administrative villages in rural areas or neighborhoods in urban areas, which comprised resident committees. In each PSU, samples of dwellings were randomly selected using mapping software named CHARLS-GIS. In total, the survey was conducted in 28 provinces, 150 countries or districts, 450 villages or urban communities, consisting of people aged 45 and over living in households, but the baseline respondents who later entered into an institution were followed. The national baseline survey was conducted from May 2011 to March 2012. The total sample households included 23,422 dwellings, and the survey finally managed to contact 17,708 individuals in 10,257 households with an overall response rate of 80.5% (26). Further details of the sample are available elsewhere (26), and data can be accessed through its official website (http://charls.pku.edu.cn/).

**Figure 1:**
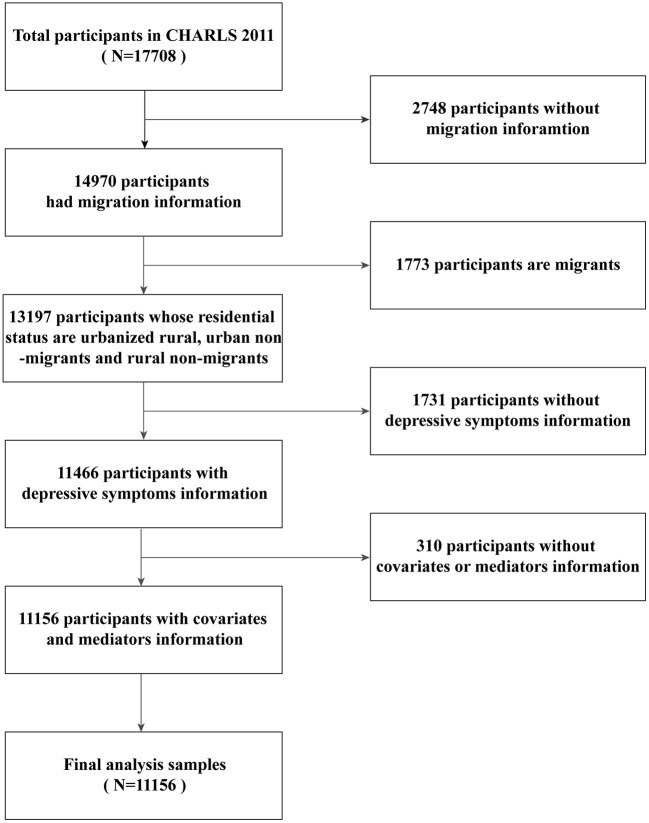
Flowchart of sampling of this study.

According to a prior study (16), we chose the CHARLS baseline survey since it contained more sufficient information on individual migration experience, while migration indicators in surveys of younger cohorts were deduced based on the 2011 survey. From the 2011 wave, we excluded 2,748 participants without information on migration and 1,731 participants without information on depressive symptoms. A total of 1,773 participants who were migrants were excluded, and 310 cases of missing covariates or mediators were also excluded. Our final sample contained 11,156 respondents with an average age of 58.91. Figure 1 presents a flowchart of the 2011 CHARLS study.

### 2.1. Measures

#### 2.1.1. Depressive symptoms

Our outcome variable was a binary measure (i.e., whether depressive symptoms were present). This study used the 10-item Center for Epidemiologic Studies Depression Scale (CESD-10) short form (27), which has satisfactory validity and reliability among the Chinese older (28) population (28, 29). The respondents were asked to rate their positive feelings, negative emotions and somatic symptoms experienced over the past week through the 10 following questions on a 4-point scale from rarely or none of the time (<1 day) to most or all of the time (5–7 days): (1) I was bothered by things that do not usually bother me; (2) I had trouble keeping my mind on what I was doing; (3) I felt depressed; (4) I felt everything I did was an effort; (5) I felt hopeful about the future; (6) I felt fearful; (7) My sleep was restless; (8) I was happy; (9) I felt lonely; (10) I could not get “going”. The depressive symptoms index was obtained from the sum of the scores of the 10 questions ranging from 0 to 30, and a higher score indicated higher depression. According to previous studies, a cutoff point of 10 was of good validity among older Chinese respondents (27, 28); consequently, respondents who scored at least 10 in this study were considered to have depressive symptoms.

#### 2.1.2. Residential status

Our independent variable was categorized as urbanized rural residents, rural non-migrants and urban non-migrants. We used both the migration information and official household registration system information (called Hukou in Chinese) to measure residential status. In detail, we first removed migrants from the sample and took non-migrants as the targeted study population to study the exogenous effect of urbanization on depressive symptoms. To do this, we used data on their birthplace, current place of residence, age at migration and duration of migration to measure their experience of migration, excluding return migrants (having a migration experience of more than 6 months outside their birthplaces) and early-life migrants (the age at migration is younger than 16). We then used Hukou status to classify the non-migrant population into three groups: (1) urbanized rural residents, referring to local urbanized residents who have realized urbanization in their towns and villages and used to hold rural Hukou when relating to the birthplace. Their lives began in the countryside, while their later lives are in the city; (2) rural non-migrants, referring to people who live in rural areas and hold rural Hukou; (3) urban non-migrants, referring to people who are born and live in urban areas and hold urban Hukou.

#### 2.1.3. Mediators

**Figure 2:**
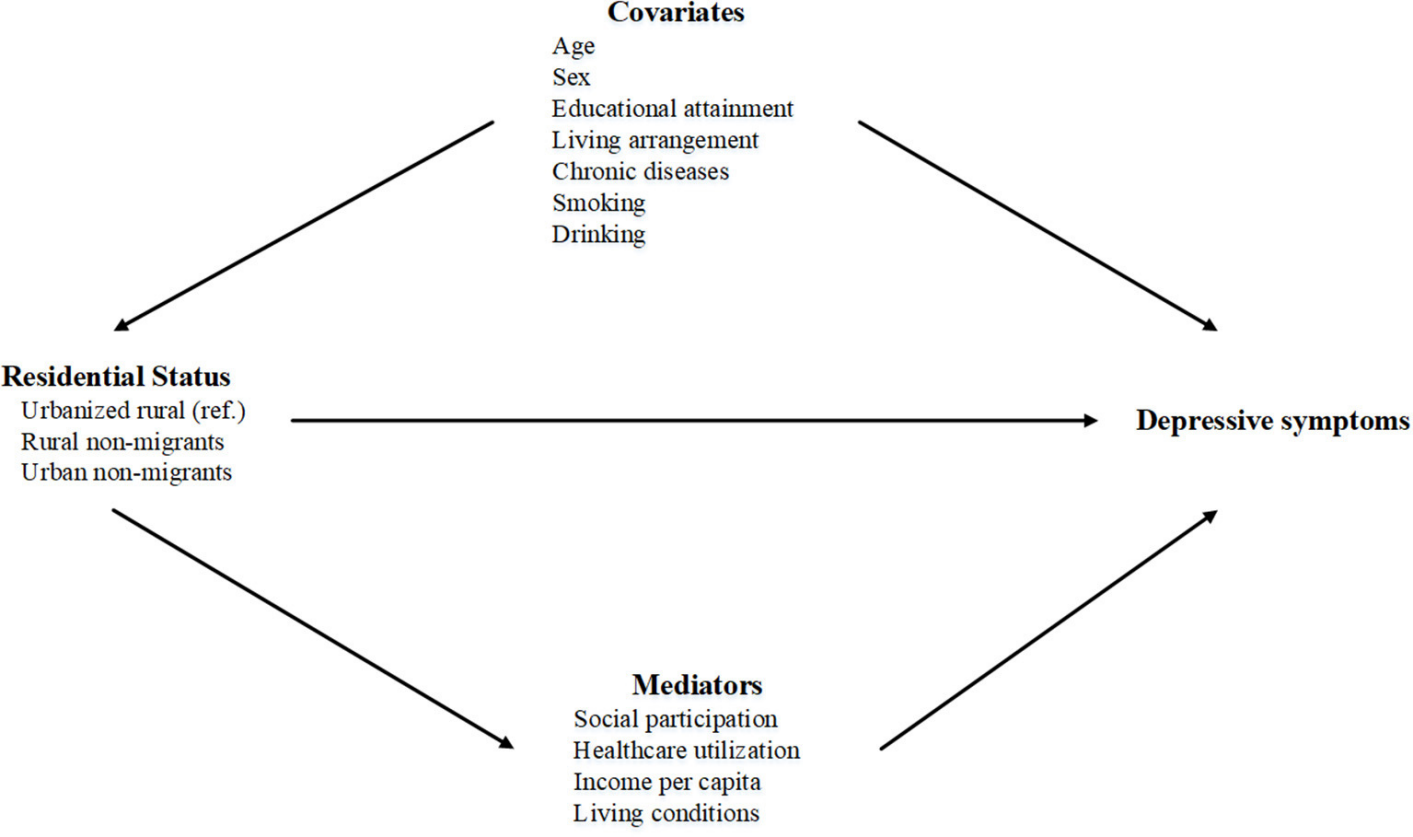
Directed acyclic graph (DAG) of the mediation model.

According to previous studies (13, 14, 20, 30–32), we considered social participation, healthcare utilization, income per capita and living conditions as mediators (see Figure 2).

##### 2.1.3.1. Social participation

In this study, social participation was a continuous variable with an aggregated count (0–11). Respondents were asked about whether they had taken part in any of the following types of social activities in the past month: (1) interacted with friends; (2) played mahjong, chess, cards or went to community clubs; (3) provided assistance to family members, friends or neighbors who do not live together for free; (4) went to a sport, social or other clubs; (5) took part in a community-related organization; (6) engaged in voluntary or charity work; (7) took care of a sick or disabled adult who does not live with the respondent for free; (8) attended an educational or training course; (9) invested in stock; (10) used the internet; (11) other, or (12) none of these. We then summed the total number of social activities one participated in from the above multiple-choice question, and the index ranged from 0 to 11, with higher scores indicating greater social participation.

##### 2.1.3.2. Healthcare utilization

This study generated a binary healthcare utilization variable (1 = yes; 0 = no). In general, healthcare utilization includes outpatient and inpatient care. In this study, we used self-reported information on whether respondents had “visited a public hospital, private hospital, public health center, clinic, or health worker's or doctor's practice, or been visited by a health worker or doctor for outpatient care in the last month” or “received inpatient care in the past year”. Based on the characteristics of the data, this study generated a healthcare utilization variable conditioned upon the occurrence of outpatient visits or inpatient visits.

##### 2.1.3.3. The income per capita

The income per capita is a continuous variable. We used the self-reported household income per capita in this study to measure the economic conditions of individuals.

##### 2.1.3.4. Living conditions

Living conditions is a continuous variable measured as an aggregated count (0–7) of seven dichotomous indicators: (1) concrete and steel/bricks and wood, (2) flushable toilet, (3) running water, (4) shower or bath facilities, (5) coal or natural gas supply, (6) telephone connection, and (7) broadband internet connection. The summed score of these seven items ranged from 0 to 7, with higher scores indicating better living conditions.

#### 2.1.4. Covariates

We included age (continuous variable), sex (female/male), educational attainment (primary school and below/junior high school and above), living arrangement (living without spouse/living with spouse), chronic diseases (1 = yes, 0 = no), smoking (1 = yes, 0 = no) and drinking (1 = yes, 0 = no).

### 2.2. Analytic strategy

In this study, descriptive statistics were used to present the characteristics of participants and the prevalence of depressive symptoms in the three groups. To address hypotheses 1(a) and 1(b), logistic regression models were used to examine the relationship between urbanization and depressive symptoms in Models 1, 2, 3, 4, and 5. In Model 1, we controlled all covariates (age, sex, educational attainment, living arrangement, chronic diseases, smoking, and drinking) to examine the joint results. In addition to the factors of Model 1, four mediators (social participation, healthcare utilization, income per capita, and living conditions) were included in Models 2–5 in turn. The analyses were performed with Stata/SE 15.0 for Windows (Stata Corp, College Station, TX, USA). In addition, to explore how social participation, healthcare utilization, income, and living conditions might mediate the association between urbanization and depressive symptoms, we applied the Structural Equation Modeling (SEM) approach to further investigate the association and address hypothesis 2(c), 2(d), 2(e), and 2(f). SEM analyses were conducted using MPlus version 8.3 (Muthén & Muthén, Los Angeles, CA, USA). A CFI > 0.90 was considered an adequate model fit (33, 34), and a *p*-value of <0.05 was considered statistically significant.

## 3. Results

### 3.1. Characteristics of participants

Among the total participants, the mean age was 58.91 years old (SD = 9.48), with 5,142 males (46.09%) and 6,014 females (53.91%). Nearly 4,262 people (38.20%) were measured as having depressive symptoms. Compared with the urbanized rural group, we found that the proportion of depressive symptoms was higher in the rural group. This rural group was older, less educated, fewer living with their spouse, and had higher prevalence of smoking or drinking but fewer chronic diseases. Rural residents were less engaged in social participation, with more healthcare utilization, lower income, and worse living conditions. Relative to urbanized rural residents, a smaller population among the urban group had depressive symptoms. The urban group participants were suffering from more chronic diseases even with younger age, better education level, and living arrangement, and less smoking and drinking. They had better living conditions, more participation in social activities, higher income, and less healthcare utilization. More details of the participants' characteristics are shown in Table 1.

**Table 1:** Characteristics of participants (*N* = 11,156).

**Characteristics**	**Urbanized-rural** **(*N* = 2,274)**	**Rural non-migrants** **(*N* = 7,135)**	**Urban non-migrants** **(*N* = 1,747)**
Depressive symptoms, *n* (%)	776 (34.12)	3,032 (42.49)	454 (25.99)
Age, mean (SD)	58.32 (9.63)	59.33 (9.50)	57.95 (9.09)
Female, *n* (%)	1,271 (55.89)	3,774 (52.89)	969 (55.47)
Primary school and below, *n* (%)	1,624 (71.42)	5,453 (76.43)	641 (36.69)
Living without spouse, *n* (%)	367 (16.14)	1,243 (17.42)	262 (15.00)
Chronic disease, *n* (%)	750 (32.98)	2,317 (32.47)	701 (40.13)
Smoking, *n* (%)	853 (37.51)	2,837 (39.76)	575 (32.91)
Drinking, *n* (%)	878 (38.61)	2,826 (39.61)	656 (37.55)
Social participation, mean (SD)	0.51 (0.87)	0.50 (0.77)	0.67 (1.17)
Healthcare utilization, *n* (%)	556 (24.45)	1,856 (26.01%)	381 (21.81)
The income per capita, mean (SD)	10,628 (29,504)	6,093 (18,533)	26,590 (54,315)
Living conditions, mean (SD)	2.80 (1.44)	2.00 (1.25)	3.76 (1.56)

**Figure 3:**
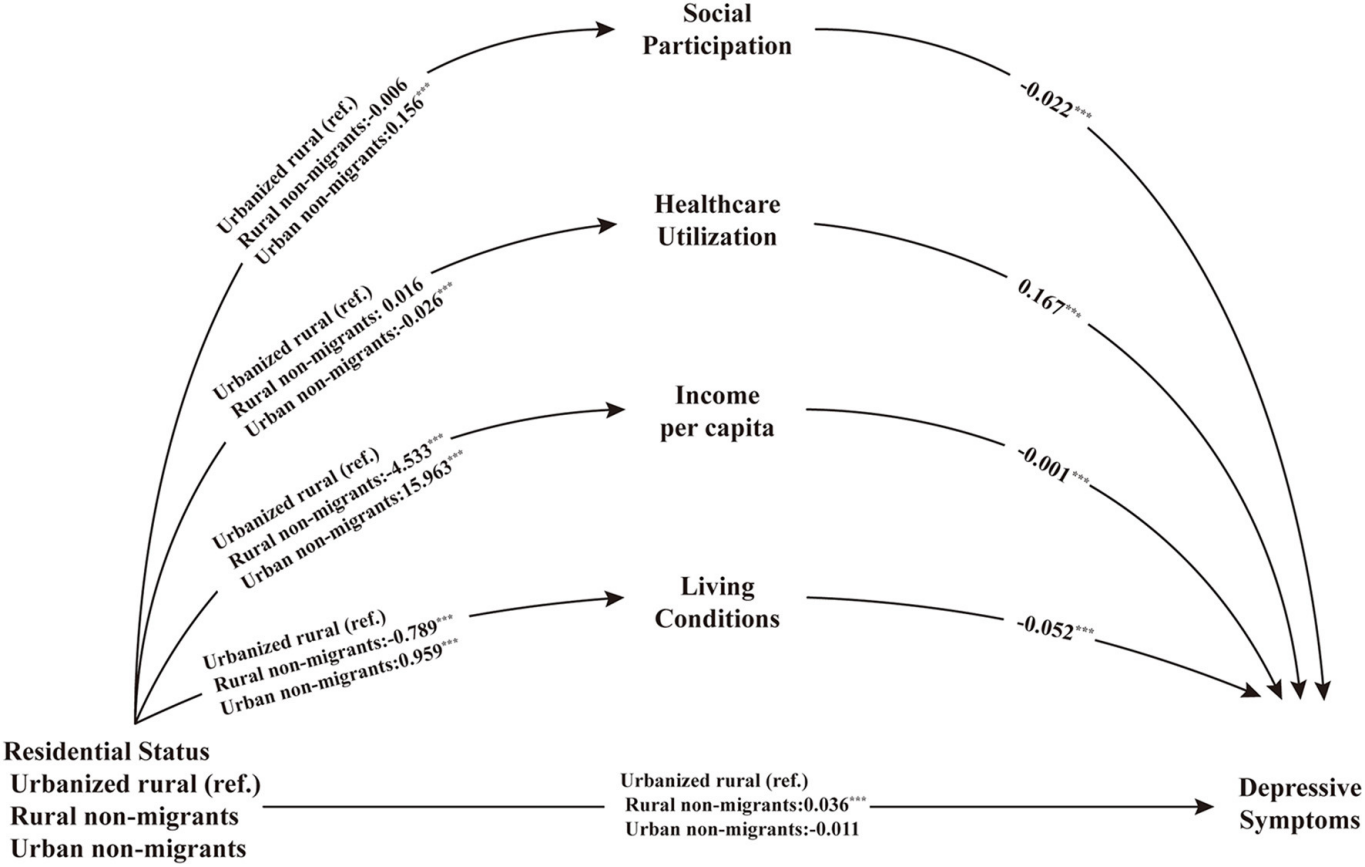
Pathways between residential status and depressive symptoms. ***p < 0.001.

The SEM on depressive symptoms showed an adequate model fit: CFI = 0.930, and the unstandardized estimates appear in Figure 3. Compared with urbanized rural residents, rural residents were associated with an increased risk of depressive symptoms (0.036, *P* < 0.002). It also predicted a lower level of income per capita (−4.533, *P* < 0.000) and worse living conditions (−0.789, *P* < 0.000), all of which were associated with an increased risk of depressive symptoms. Relative to urbanized rural residents, urban residents were not directly associated with depressive symptoms. Urban residents predicted higher engagement in social participation (0.156, *P* < 0.000), less healthcare utilization (−0.026, *P* < 0.047), higher income per capita (15.963, *P* < 0.000), and better living conditions (0.959, *P* < 0.000), all of which were negatively associated with depressive symptoms except healthcare utilization. The direct and indirect effects of urbanization on depressive symptoms are reported in **Table 3**. Rural residents had both direct and indirect effects on depressive symptoms, but we observed no direct effects in the urban group.

### 3.2. Logistic regression of the association between residential status and the risk of depressive symptoms

Table 2 shows the results of the multivariate logistic regression analysis between residential status and depressive symptoms. According to Model 1, compared with the urbanized-rural group, the rural group was more likely to have depressive symptoms (OR = 1.42, 95% CI: 1.28, 1.57). The value of OR changed slightly after including the factors of social participation and healthcare utilization in Model 2 and Model 3. After adjusting for income per capita in Model 4, the OR decreased from 1.42 (95% CI = 1.28, 1.57) to 1.38 (95% CI = 1.25, 1.53), and after adjusting for living conditions in Model 5, the odds ratio changed from 1.38 (95% CI = 1.25, 1.53) to 1.19 (95% CI = 1.07, 1.32). Relative to the urbanized-rural group, the urban group was associated with a decreased risk of depressive symptoms (OR = 0.79, 95% CI: 0.68, 0.92) when adjusting for all covariates in Model 1, and the association remained after controlling for social participation (OR = 0.80, 95% CI: 0.69, 0.93) in Model 2 and healthcare utilization in Model 3 (OR = 0.81, 95% CI: 0.70, 0.94). We found that after controlling for income per capita (Model 4) and living conditions (Model 5), this association was not statistically significant, which indicated that this difference may be explained by income and living conditions.

**Table 2:** Logistic regressions of the association between residential status and the risk of depressive symptoms (*N* = 11,156).

**Characteristics**	**Model 1^a^ OR (95% CI)**	**Model 2^b^** **OR (95% CI)**	**Model 3^c^ OR (95% CI)**	**Model 4^d^** **OR (95% CI)**	**Model 5^e^ OR (95% CI)**
Residential status	–	–	–	–	–
Urbanized rural	Reference	Reference	Reference	Reference	Reference
Rural non-migrants	1.42 (1.28, 1.57)	1.42 (1.28, 1.57)	1.42 (1.28, 1.57)	1.38 (1.25, 1.53)	1.19 (1.07, 1.32)
Urban non-migrants	0.79 (0.68, 0.92)	0.80 (0.69, 0.93)	0.81 (0.70, 0.94)	0.88 (0.76, 1.02)	1.01 (0.87, 1.18)

a Model 1 adjusted for residential status, age, sex, educational attainment, living arrangement, chronic diseases, smoking, and drinking.

b In addition to factors in model 1, model 2 was adjusted for social participation.

c In addition to factors in models 1 and 2, model 3 was adjusted for healthcare utilization.

d In addition to factors in models 1, 2, and 3, model 4 was adjusted for income per capita.

e Fully adjusted model 5 comprised the factors in models 1, 2, 3, and 4 plus living conditions.

### 3.3. Pathways between residential status and depressive symptoms

The SEM on depressive symptoms showed an adequate model fit: CFI = 0.930, and the unstandardized estimates appear in Figure 3. Compared with urbanized rural residents, rural residents were associated with an increased risk of depressive symptoms (0.036, *P* < 0.002). It also predicted a lower level of income per capita (−4.533, *P* < 0.000) and worse living conditions (−0.789, *P* < 0.000), all of which were associated with an increased risk of depressive symptoms. Relative to urbanized rural residents, urban residents were not directly associated with depressive symptoms. Urban residents predicted higher engagement in social participation (0.156, *P* < 0.000), less healthcare utilization (−0.026, *P* < 0.047), higher income per capita (15.963, *P* < 0.000), and better living conditions (0.959, *P* < 0.000), all of which were negatively associated with depressive symptoms except healthcare utilization. The direct and indirect effects of urbanization on depressive symptoms are reported in Table 3. Rural residents had both direct and indirect effects on depressive symptoms, but we observed no direct effects in the urban group.

**Table 3:** Direct effect, indirect and total effect of residential status on depressive symptoms (*N* = 11,156).

**Characteristics**	**Depressive symptoms**		
	**Direct effects**	**Indirect effects**	**Total effects**
Urbanized rural	Reference	Reference	Reference
Rural non-migrants	0.036^***^ (0.014, 0.058)	0.048^***^ (0.041, 0.055)	0.084^***^ (0.061, 0.105)
Urban non-migrants	−0.011 (−0.040, 0.018)	−0.071^***^ (−0.081, −0.060)	−0.081^***^ (−0.110, −0.053)

*** P < 0.001.

## 4. Discussion

Using large nationally representative and population-based data, this study was the first to explore the potential mechanism of urbanization and depressive symptoms independent of health selective migration among Chinese middle-aged and older adults. Overall, we found that urbanization was significantly associated with a decreased risk of depressive symptoms. These associations were more likely to be mediated indirectly through social participation, income per capita and living conditions.

Evidence supports that the association between urbanization and depressive symptoms is controversial because of different progress in urbanization and differences in culture, economic development, and environmental factors. On the one hand, the fact of this finding is contrary to the common belief that most people believe individuals living in the countryside are less prone to depression (13), which could be explained by several reasons. In most high-income countries, compared with urban areas, rural residents who haven't experienced urbanization are exposed to less economic stress or environmental hazards (16, 17) and the violent crime rate is lower (35), resulting in a lower risk of depression. In low-income countries, compared to urban residents, rural residents are disadvantaged in socioeconomic status and access to health services, especially mental health services (12). Moreover, urbanization increases the prevalence of depression in most low-income countries, as in the context of India, where unplanned urbanization progresses with the growth of squatters, slum settlements, and apparent poor living conditions (19). On the other hand, previous studies in the context of the United States, where urbanization has orderly coordinated population, land, and socioeconomic subsystems, can substantially lower rates of depression with better urban physical and socioeconomic environments (32). Consistent with findings in the United States, our findings also found a beneficial effect of planned urbanization on mental health promotion. Our results showed that the urban group was associated with a decreased risk of depressive symptoms compared with the urbanized rural group, which supported the life course theory, indicating that the accumulation of adverse events in the life course may result in poor health in old age (36). Unlike other countries, China's rapid urbanization has been largely encouraged by the government which helps to avoid the negative effects of urbanization (16, 18). Furthermore, urbanized rural residents might be advantaged in socioeconomic conditions, mental health services and social connection with a lower rate of violent crime due to the expansion of housing and infrastructure which are beneficial for older adults.

Moreover, we found that although urbanization could compensate for the negative impact of depression from disadvantaged early life conditions, it could not completely reverse the health differentials in early life and eliminate the gap between urbanized rural residents and urban residents. Two different hypotheses related to the modifiable or unmodifiable health consequences of adverse events have been proposed. From the embedding mechanism perspective, many diseases developed later in later life originate through epigenetic marks, post-translational modifications, and tissue remodeling caused by adverse events in early life (37), indicating that exposure to adverse events is not modifiable by subsequent wellbeing and that early life is a key period for interventions to reduce future health disadvantages. Another hypothesis suggests that the association of early adversities with health outcomes in later life may be explained by chains of risk (38), which indicates that urbanization may compensate for the negative impact of disadvantaged early life conditions on depression.

Concerning the association between urbanization and depressive symptoms, the primary mediators were social participation, income and living conditions. First, as the urban scaling theory suggests, city environments and urbanization can naturally provide greater social stimulation and connections (32), and engagement in more social participation might improve physical function (39) and cognitive function (40), which is positively related to lower depressive symptoms. This evidence supports our findings that urbanization may create expanding opportunities for social participation, which helps to decrease the risk of depressive symptoms. Second, urbanization may lower the risk of depressive symptoms through the improvement of income level, which is in line with previous evidence (41). As one of the most important indicators of socioeconomic status, income is strongly associated with depression among middle-aged and older adults in China (31, 42, 43). Third, better living conditions may be related to high-quality houses (44) with improved infrastructure and indoor facilities (30), which may be more suitable for middle-aged and older adults to live in and help lower their risk of depressive symptoms. These results may suggest that the mediating effect is a consequence of a planned urbanization process, associated infrastructure improvement, and better social connections.

### 4.1. Limitations

This study used a large nationally representative sample and isolated the impact of urbanization on depressive symptoms independent of health-selective migration or salmon bias by identifying a population in China who were transformed from local villagers to urban citizens without being influenced by migration. Another key strength of this study is the detection of the mediating mechanisms by which urbanization influenced depressive symptoms. However, this study also had several limitations. First, due to limited data, the measurement of some variables may result in bias in the results when exploring the possible underlying mechanism. For example, outpatient services might be underestimated because some participants may have had no outpatient visits before the survey. Moreover, not including other variables related to depression might have resulted in residual confounding that could bias estimates (45). Second, this study could not rule out a survival effect since healthier people tend to live longer, so we may have underestimated the differences in health conditions, such as depressive symptoms and chronic diseases. Third, limited by data acquisition, urban factors negatively related to health were not included, such as air pollution. Therefore, further studies are needed to confirm the results. Fourth, this survey is a cross sectional study and it measured the depressive symptoms by self-reported data, which may lead to recall bias (10).

### 4.2. Conclusions

To conclude, our study found that urbanization in China, almost effectively managed by the state, had an independent impact on decreased depressive symptoms among middle-aged and older adults after isolating the effect of health selective migration. Improvements in underlying mechanisms, including living conditions, social participation, and income per capita, are likely to be the main drivers that benefit mental health among middle-aged and older populations. Additionally, our comparison of depressive symptoms across urbanized rural residents and urban residents who shared similar later urban lives while having different early life circumstances revealed that early adverse events might have the potential to be modified by prevention through socioeconomic factors in later life but might not be fully modifiable. Our findings indicated that planned urbanization may benefit health and wellbeing, and a human-oriented urbanization pattern with orderly industrial upgrading, employment transfer, and population agglomeration may provide support for building a healthy society.

## Data availability statement

Publicly available datasets were analyzed in this study. This data can be found here: http://charls.pku.edu.cn/.

## Ethics statement

The studies involving human participants were reviewed and approved by the Biomedical Ethics Review Committee of Peking University. The patients/participants provided their written informed consent to participate in this study.

## Author contributions

YLu conceived and designed the study. CH and YLu did the initial analysis and supervised data analysis. CH wrote the first draft of the paper. YLu, XX, and XN critically revised the first draft. JL, DQ, YY, and YLi did a thorough language check through the manuscript. CH, XX, JL, XN, DQ, YY, YLi, and YLu reviewed the manuscript. All authors contributed to the article and approved the submitted version.
